# ﻿Two new species of the genus *Thereuopoda* Verhoeff, 1904 (Scutigeromorpha, Scutigeridae) from Sichuan and Hainan Provinces, China

**DOI:** 10.3897/zookeys.1264.165241

**Published:** 2025-12-19

**Authors:** Jie-Hong Ji, Chen-Yang Shen, Yi-Xin Gao, Hui-Yuan Wu, Dan-Na Yu, Sheng-Long Liu, Jia-Yong Zhang

**Affiliations:** 1 College of Life Sciences, Zhejiang Normal University, Jinhua, 321004, Zhejiang Province, China; 2 Key Lab of Wildlife Biotechnology, Conservation and Utilization of Zhejiang Province, Zhejiang Normal University, Jinhua, 321004, Zhejiang Province, China; 3 Longquan Conservation Center of Qianjiangyuan-Baishanzu National Park, Longquan, 323000, Zhejiang Province, China

**Keywords:** Centipede, molecular phylogeny, morphology, scutigeromorph, taxonomy

## Abstract

Scutigeromorph centipedes are morphologically distinctive myriapods that have historically received limited scientific attention, particularly in China. The taxonomy of the genus *Thereuopoda* Verhoeff, 1904 has remained largely unresolved since the late 1970s, and recent molecular evidence has indicated the presence of cryptic species within this group. In this study, we describe two new species, *T. kaijiangensis***sp. nov.** Ji, Shen & Zhang from Kaijiang County, Dazhou City, Sichuan Province, China, and *T. edgecombei***sp. nov.** Ji, Liu & Zhang from Chengmai County, Hainan Province, China. Comprehensive morphological descriptions and detailed illustrations are provided for both species, along with an updated diagnostic key to all currently recognized *Thereuopoda* species in China. Phylogenetic analyses conducted using both maximum-likelihood and Bayesian-inference methods consistently support the monophyly of the genus *Thereuopoda* and further validate the taxonomic distinctness of the two newly described species.

## ﻿Introduction

Scutigeromorpha, the most morphologically distinct of the five centipede orders, is characterized by a suite of unique morphological features, including multisegmented tarsi, a domed head capsule, compound eyes, and dorsally positioned spiracles located on the tergites (Perez-Gelabert and Edgecombe 2013). The family Scutigeridae constitutes the largest family in this order, comprising approximately 18 genera and 90 species (Porta and Giribet 2024). Two subfamilies are currently recognized in Scutigeridae: Scutigerinae Leach, 1814 and Thereuoneminae Verhoeff, 1905. The genus *Thereuopoda* Verhoeff, 1904, distributed across East and Southeast Asia, is classified within the subfamily Thereuoneminae (Bonato et al. 2016).

The earliest *Thereuopoda* species discovered in Hong Kong is *Thereuopoda clunifera* (Wood, 1862), which was originally placed in the genus *Cermatia* (Wood 1862). Subsequently, further taxonomic and faunistic studies were conducted by Meinert (1886), Haase (1887), and Pocock (1895). The genus *Thereuopoda* was originally established as a subgenus by Verhoeff (1904) and comprised four species, *T. rubrolineata* Verhoeff, 1904, *T. longicornis* (Fabricius, 1793), *T. clunifera* (Wood, 1862), and *T. amokiana* Verhoeff, 1904. Subsequently, Verhoeff (1905) elevated *Thereuopoda* to generic rank and subdivided it into three subgenera: *Orthothereua* Verhoeff, 1905, *Thereuopodasensu stricto* Verhoeff, 1904, and *Microthereua* Verhoeff, 1905. During the first half of 20^th^ century, numerous new species of *Thereuopoda* were described by Chamberlin (1944) and Verhoeff (1925, 1936, 1937, 1939, 1942, 1943, 1944), including *Thereuopoda nivicomes* Verhoeff, 1942 from China (“Quellgebiet des Yangtsekiang bei Dji-tu im Hzifan-Bergland”). Verhoeff (1936) also established a new genus *Teleotelson* Verhoeff, 1936 for two species previously misassigned to *Thereuonema* by Silvestri (1924). In the 1950s, Chamberlin and Wang reported *T. clunifera* from Taiwan Island (Chamberlin and Wang 1952; Wang 1955, 1956, 1959). In 1979, the taxonomy of *Thereuopoda* underwent comprehensive revision (Würmli 1979). The genus *Telotelson* and the subgenus Orthothereua were recognized as junior synonyms of *Thereuopoda* and many species described by early taxonomists were treated as junior synonyms of *T. longicornis* and *T. clunifera*. The taxonomic status of *Thereuopoda chinensis* Verhoeff, 1905 and its subgenus Microthereua remains uncertain due to the absence of examined type material. Since 1979, only a few publications have reported records of *Thereuopoda* species (Stoev 2002; Stoev and Geoffroy 2004; Li 2007; Xu et al. 2013; Dunlop et al. 2017; Lei et al. 2021; Huang et al. 2023). To date, three valid species of *Thereuopoda* have been documented in China, T. (M.) chinensis, *T. clunifera*, and *T. longicornis*. The generic placement of two poorly known taxa—*Scutigera complanata* Haase, 1887 and *Scutigera sinuata* Haase, 1887—remains uncertain (Haase 1887).

**Figure 1.**
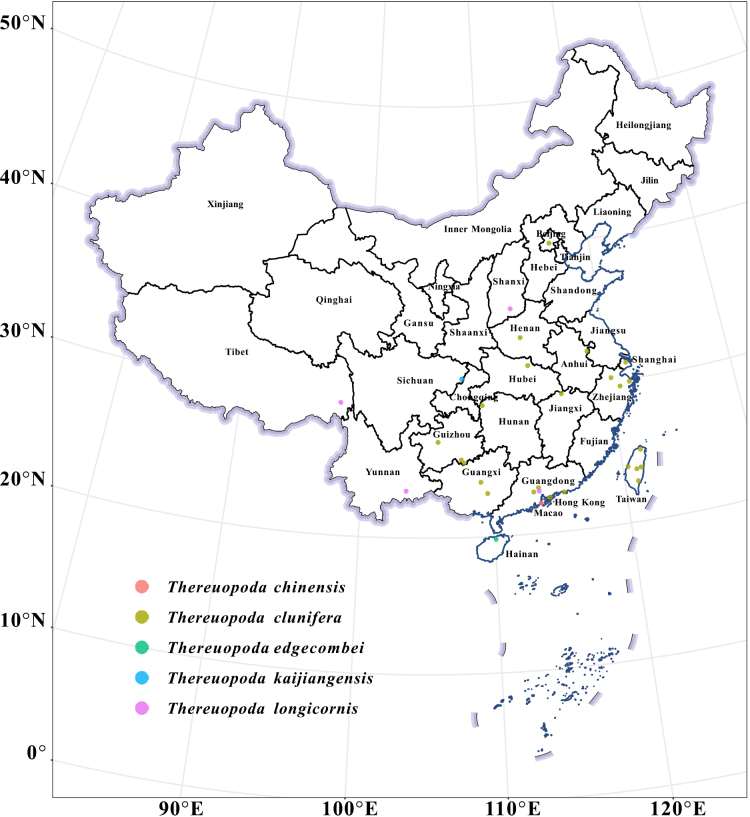
The distribution of the five known *Thereuopoda* species in China. *T. chinensis*: Macao. *T. clunifera*: Taitung County, Haulien County, Nantou County, Changhua County and Taipei City, Taiwan Province; Hong Kong; Nanjing City, Jiangsu Province; Hangzhou City, Shaoxing City and Ningbo City, Zhejiang Province; Guangzhou City, Foshan City and Shanwei City, Guangdong Province; Guigang City and Laibin City, Guangxi Province; Pingdingshan City, Henan Province; Beijing Municipality; Shanghai Municipality; Jiujiang City, Jiangxi Province; Bijie City and Libo County, Guizhou Province; Longshan County, Hunan Province. Suizhou City, Hubei Province. *T. kaijiangensis* sp. nov.: Dazhou city, Sichuang Province. *T. edgecombei* sp. nov.: Chengmai County, Hainan Province. *T. longicornis*: Guangzhou City, Guangdong Province; Qamdo City, Tibet; Menzi County, Yunnan Province; Qinshui County, Shanxi Province.

The remarkable diversity and complexity of China’s zoogeographical and geological characteristics have contributed to an exceptionally rich faunal assemblage. Recent studies have revealed cryptic species within the genus *Thereuopoda* and high diversity in *Thereuonema tuberculata* (Wood, 1862) through molecular data analyses (Yang et al. 2022; Manivannan et al. 2024; Ji et al. 2025). In this study, two new species—*Thereuopoda kaijiangensis* sp. nov. Ji, Shen & Zhang and *Thereuopoda edgecombei* sp. nov. Ji, Liu & Zhang—are described, and their phylogenetic relationships are analyzed. The distribution of the five known *Thereuopoda* species in China is shown in Fig. 1.

## ﻿Materials and methods

### ﻿Specimens

All samples were collected using the button tube method and preserved in 80% ethanol in 2023 and 2024. Specimens were examined under an Olympus SZX16 stereomicroscope (Olympus Corporation, Tokyo, Japan) and a NOVEL DN-401 biological microscope (Ningbo Yongxin Optics Co. Ltd, Ningbo, China). Images were captured with an Olympus DP73 digital camera (Olympus Corporation, Tokyo, Japan). The raw photographs were processed through image alignment and focus-stacking in Affinity Photo v. 2.6.0 (Serif Ltd, Nottingham, UK) to generate composite images, which were subsequently refined with Adobe Photoshop CC 2019 (Adobe Systems Inc., Los Angeles, CA, USA). Type specimens are deposited in the Animal Herbarium at Zhejiang Normal University, Jinhua, China (**ZJNU**).

Morphological terminology follows that of Edgecombe and Giribet (2006) and Edgecombe (2011). The measurement protocol for the female gonopod follows Würmli (1973). Tergites are abbreviated as TT1–TT8.

### ﻿Molecular analyses

Six molecular markers (18S rRNA, 28S rRNA, H3, 16S rRNA, 12S rRNA, and COX1), previously used in scutigeromorph phylogenetic analyses (Giribet and Edgecombe 2013; Manivannan et al. 2024), were sequenced to assess the phylogenetic position of the two new species. Two legs of the holotype were used to extract genomic DNA with the Ezup Column Animal Genomic DNA Purification Kit (Sangon Biotech Co., Shanghai, China). Genomic DNA extraction and subsequent library preparation were performed by BerryGenomics (Beijing, China) for next-generation sequencing (NGS). Raw NGS data were quality-filtered and de novo assembled using MEGAHIT v. 1.2.9 (Li et al. 2015). The nuclear ribosomal genes 18S rRNA and 28S rRNA were obtained using Barrnap v. 0.9 (Seemann 2013). The histone H3 gene was identified using HMMER v. 3.4 (Eddy 2011). The three mitochondrial genes (16S rRNA, 12S rRNA, and COX1) were obtained following the protocol described in Ji et al. (2025). All newly generated sequences have been deposited in GenBank (Suppl. material 1). The final data used for phylogenetic analyses are summarized in Suppl. material 1.

Six genes were aligned using MAFFT v. 7.475 (Katoh and Standley 2013), and the resulting alignments were trimmed with Gblocks v. 0.91b (Castresana 2000). The individual gene alignments were concatenated using PhyloSutie v. 1.2.2 (Zhang et al. 2020). Partitioning schemes and best-fitting models for each partition were determined using PartitionFinder v. 2.2.1 (Lanfear et al. 2012) (Suppl. material 2). The maximum-likelihood (ML) tree was inferred using RaxML v. 8.2.0 (Stamatakis 2014) with 1,000 bootstrap replicates. The Bayesian-inference (BI) tree was conducted in MrBayes v. 3.2.7a (Ronquist et al. 2012) with 10 million MCMC generations, sampling every 1,000 generations and applying a burn-in of 25%. The resulting phylogenetic trees were visualized and edited using Affinity Photo v. 2.6.0 (Serif Ltd, Nottingham, UK). Genetic distances for COX1 were calculated in MEGA v. 11 (Tamura et al. 2021) using the Kimura 2-parameter model.

## ﻿Results

### ﻿Order Scutigeromorpha Pocock, 1895

**Family Scutigeridae Gervais, 1837**

**Subfamily Thereuoneminae Verhoeff, 1925**

**Genus *Thereuopoda* Verhoeff, 1904**

#### ﻿ Thereuopoda kaijiangensis

Taxon classificationAnimalia

Ji, Shen & Zhang sp. nov.

3E868653-4C0F-5943-9D1A-F943539472D8

https://zoobank.org/41807141-54CA-4320-B932-DFA2BC1656C8

**Figure 2.**
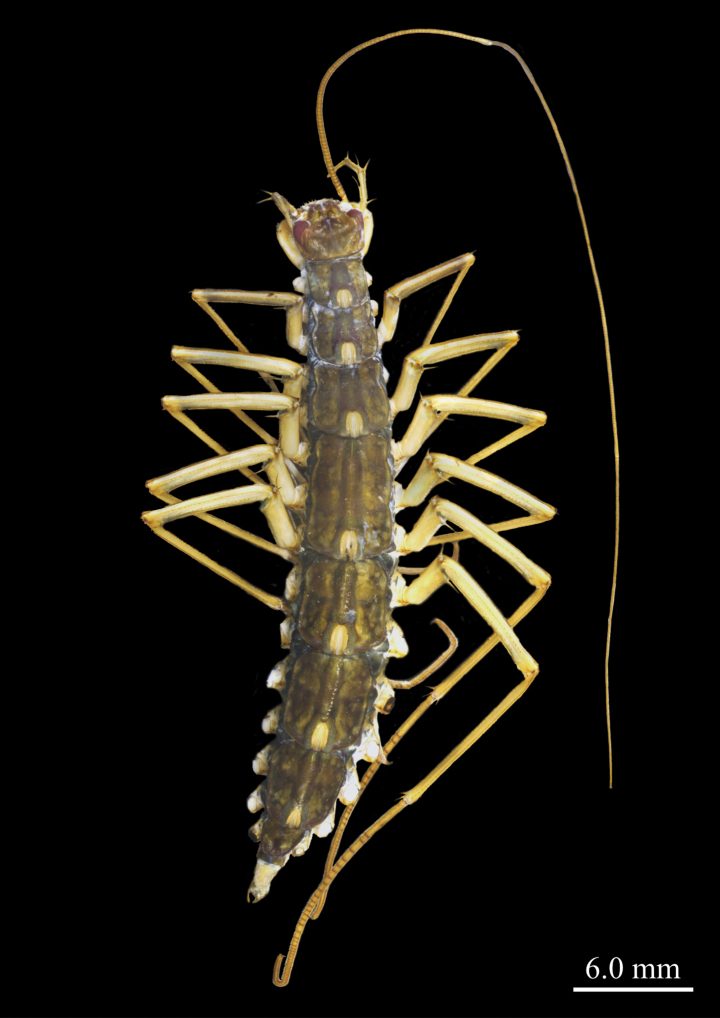
*Thereuopoda kaijiangensis* sp. nov., paratype female. Scale bars: 6.0 mm.

**Figure 3.**
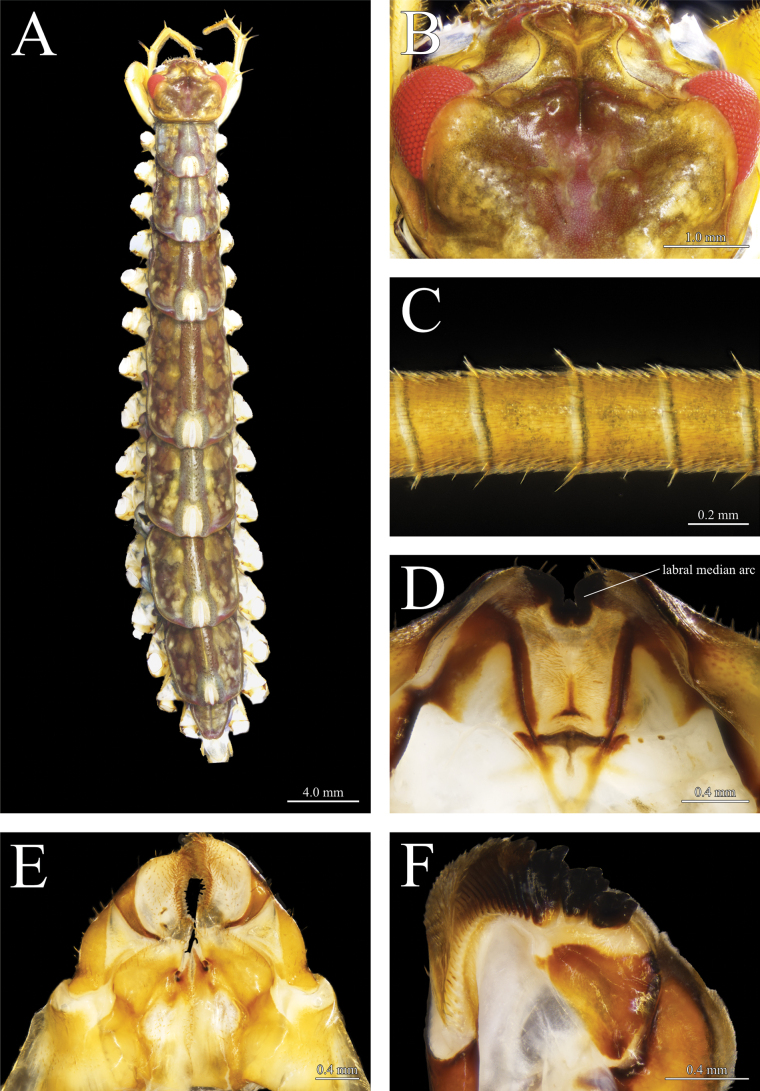
*Thereuopoda kaijiangensis* sp. nov., holotype male. **A.** Habitus, dorsal view; **B.** Head capsule; **C.** First flagellum of antenna; **D.** Epipharynx; **E.** First maxillae; **F.** Mandible. Scale bars: 4.0 mm (**A**); 1.0 mm (**B**); 0.2 mm (**C**); 0.4 mm (**D–F**).

**Figure 4.**
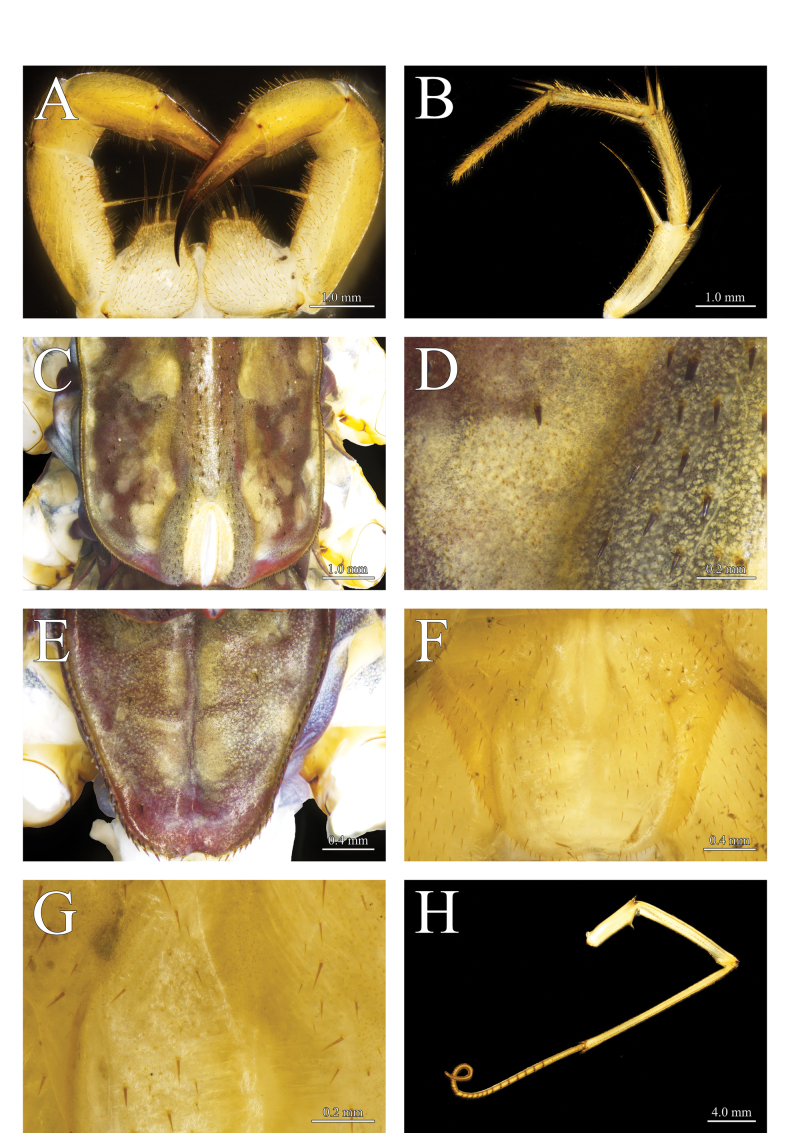
*Thereuopoda kaijiangensis* sp. nov., holotype male. **A.** Forcipular segment; **B.** Second maxilla; **C.** Tergite 6; **D.** Details of tergite 6; **E.** Tergite 8; **F.** Sternite 15; **G.** Details of sternite 15; **H.** Leg 10. Scale bars: 1.0 mm (**A–C**); 0.2 mm (**D, G**); 0.4 mm (**E, F**); 4.0 mm (**H**).

**Figure 5.**
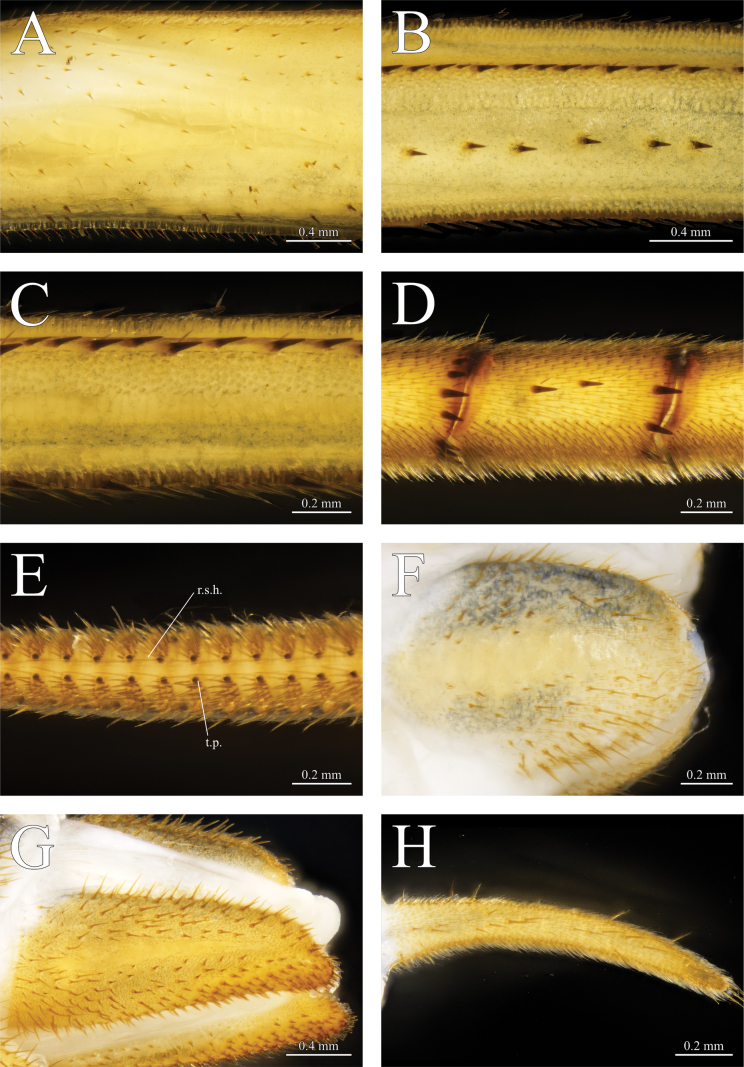
*Thereuopoda kaijiangensis* sp. nov., holotype male. **A.** Leg 10 prefemur; **B.** Leg 10 femur; **C.** Leg 10 tibia; **D.** Leg 10 tarsus I; **E.** Leg 10 tarsus II, ventral view, showing resilient sole hairs (r.s.h.) and tarsal papillae (t.p.); **F.** Male subanal plate; **H.** First male gonopod. Paratype female. **G.** Female subanal plate. Scale bars: 0.4 mm (**A, B, G**); 0.2 mm (**C–F, H**).

**Figure 6.**
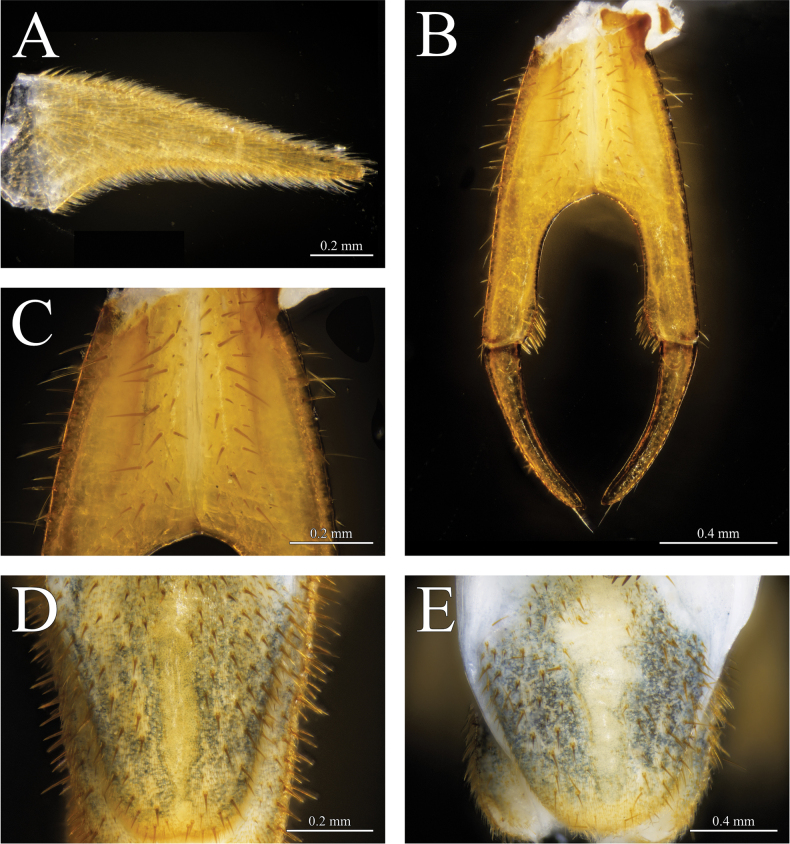
*Thereuopoda kaijiangensis* sp. nov., holotype male. **A.** Second male gonopod; **B.** Female gonopod; **C.** Proarthron of female gonopod; **E.** Male telson. Paratype female. **D.** Female telson. Scale bars: 0.2 mm (**A, C, D**); 0.4 mm (**B, E**).

**Figure 7.**
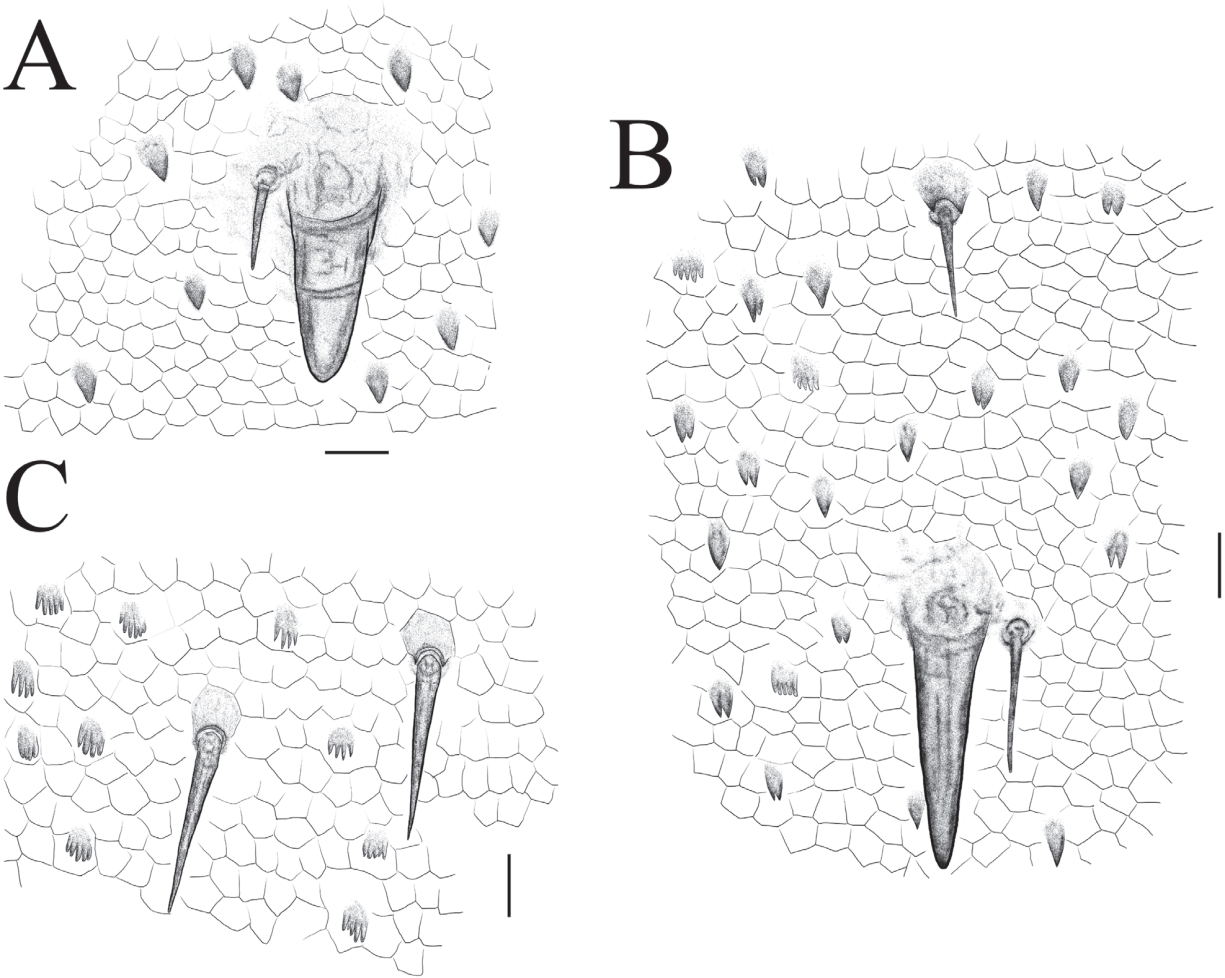
*Thereuopoda kaijiangensis* sp. nov., paratype female. **A.** Tergite 6; **B.** Tergite 8; **C.** Sternite 15. Scale bars: 20 μm.

Figs 2, 3, 4, 5, 6, 7; Tables 1, 2

##### Type material.

***Holotype*.** China • Male; Sichuan Province, Dazhou City, Kaijiang County, Yongxing Town; 31°08'N, 107°53'E; 11 May 2024; Jiehong Ji leg.; ZJNU SCKJ102. ***Paratype*.** China • 3 females; same data as for holotype; ZJNU SCKJ129–131.

##### Diagnosis.

*Thereuopoda* with body length up to 40 mm (Fig. 2). Head capsule yellow with a reddish-brown medial patch surrounded by dark-brown pigment; radiating network of dark-brown pigment on the posterolateral part of head capsule (Fig. 3A, B). Tergites pale yellow with three longitudinal dark-brown stripes; transverse projections between central and lateral stripes; a narrow yellow stripe in the middle of central stripe (Figs 3A, 4C). Stoma saddles blue-gray; reddish-purple pigment present laterally (Fig. 4C). Posterior margin of tergites unevenly rounded. Spiracle on T6 about 75–85% length of the stoma saddle. Posterior margin of T8 with slight median concavity (Fig. 4E). Leg 15 2.85–3.14× body length. Tibial spine-bristles 0/2 on leg 1. Maximum length of female gonopod 2.25–2.6× maximum width (Fig. 6B, C). Hairs absent in the central area of proarthron; angle at median distal end of proarthron about 130° (Fig. 6C). Sinus between mesarthron broadly parabolic (Fig. 6B). Posterior termination of female subanal plate pointed or with process (Fig. 5G). Length of first male gonopod 1.5× second male gonopod (Figs 5H, 6A).

##### Description.

Length up to 39 mm in largest female, 40 mm in largest male.

**Colour**: head capsule yellow with a reddish-brown patch medially surrounded by dark brown pigment; radiating network of dark brown pigment in the posterolateral part of head capsule (Fig. 3A, B). Compound eyes bright red. Tergites pale yellow with three longitudinal dark-brown stripes; transverse projections between central and lateral stripes; a narrow yellow stripe in the middle of central stripe (Figs 3A, 4C). Stoma saddles blue-gray; reddish-purple pigment present laterally. Leg yellow with blackish-green pigment on prefemur and femur (Fig. 4H). Sternites and female gonopod yellow (Figs 4F, 6B).

**Head capsule**: anterior projection of sutures with divergent posterior part; lateral edge blunt and rounded (Fig. 3B). Posteromedian impression moderately deep. Antenna 1.57–1.8× body length. First flagellum with 73–78 articles; ring-like articles with dense hairs and a few setae forming a single whorl surrounding distal end of article (Fig. 3C).

**Epipharynx**: labral median arc 1.94–2.28× depth of labral mid-piece tooth (Fig. 3D).

**Mandible**: three teeth, all smooth-surfaced and tricuspid (Fig. 3F). Nearly 23 pectinate lamellae on gnathal edge.

**First maxilla**: inner margin of distal article of telopodite with dense, brush-like setae (Fig. 3E). Short, simple setae on ventral surface of coxal process.

**Second maxilla**: prefemur with dorsal and ventral spine-bristles; femur with four spine-bristles; tibia with two dorsal spine-bristles (Fig. 4B).

**Forcipular segment**: coxa nearly as long as wide; anterior margin bearing four subequal long spine-bristles (Fig. 4A).

**Tergites**: posterior margin of tergites unevenly rounded (Fig. 4C). Borders of TT6–8 with heavy spines in saw-like fringe. Length: median-wide ratio in T4 1.46–1.6. Stoma saddles strongly vaulted. Spiracles very long, spiracle on T6 about 75–85% length of the stoma saddle. Posterior margin of T8 with slight median concavity (Fig. 4E). Number of spines on stoma saddles and tergites as shown in Table 1. Unpaired large spines on TT2–8, each paired with slender, needle-like bristles (*Tastborsten*) (Figs 4D, 7A, B). Isolated *Tastborsten* with short, paired spines at the bases on all tergites; these spines joined at a common base. Basal spines 10–20% as long as associated bristle. All tergites with spiculae and spinulae. Short, triangular spiculae separated by several polygonal scales that lack spiculae.

**Table 1.** Number of spines on stoma saddles and whole tergites of *Thereuopoda kaijiangensis* sp. nov.

Tergites	Stoma saddles	Whole tergites
1	0	0
2	0–18	13–39
3	50–79	120–157
4	56–94	220–267
5	70–90	193–197
6	58–86	149–186
7	24–45	81–114
8	–	3–4

**Legs**: tarsus I and II segmentation as shown in Table 2. Leg 15 2.85–3.14× body length. Prefemoral spine-bristles in a 2/1 pattern on legs 1–15; femoral spine-bristles 1/2 on all legs; tibial spine-bristles 0/2 on leg 1–2, 1/2 on leg 3–15. Spine rows on prefemur, femur and tibia with spine–setae pairing (Fig. 5A–C). Tarsus I of leg 4–14 with spines (Fig. 5D). Tarsus II of legs 1–14 with paired and equally sized tarsal papillae on successive segments except for first and last few segments on each leg (Fig. 5E). Resilient sole hairs originating near posteromedial edge of tarsal papilla, extending to the succeeding segment. Dense tuft of setae on ventrolateral side of tarsus II.

**Table 2.** Number of segments in Tarsus I and Tarsus II of *Thereuopoda kaijiangensis* sp. nov.

Leg	Tarsus I	Tarsus II
1	24–26	54–64
2	20–21	54–63
3	17–18	52–54
4	17–18	51–54
5	16–21	45–50
6	14–16	46–49
7	13–16	45–50
8	14–19	40–51
9	15–16	43–49
10	14–16	48–49
11	11–15	49–52
12	14–16	46–54
13	13–14	54–55
14	15–23	56–60

**Sternites**: median embayment in posterior margin of sternites lacking or shallow (Fig. 4F). Longitudinal median furrow observable passing through entire length of the sternites. Minute multifurcating spinulae and scattered setae with paired short spines on all sternites (Figs 4G, 7C).

**Female**: gonopod with maximum length 2.25–2.6× maximum width. Longitudinal median suture in proarthron complete (Fig. 6B, C). Lateral margins of proarthron and mesarthron posteriorly divergent. Subtriangular depression on proarthron without setae. Only setae in the middle area of proarthron; hairs absent (Fig. 6C). Angle at median distal end of proarthron about 130°. Proarthron 1.12–1.19× length of mesarthron. Distomedial corner of mesarthron with a cluster of 10–14 setae. Sinus between mesarthron broadly parabolic. Width of mesarthron 0.44–0.56 times maximum width of sinus. Proarthron + mesarthron 1.86–2.14× length of metarthron. Outer margin of metarthron uniformly curved. Subanal plate with ventral margin straighter than curved dorsal margin, posterior termination pointed or with process; maximum length 1.96–2.48× maximum height; smooth, non-setose band along middle of subanal plate; setae of varied size on outer surface of subanal plate between which are slender, curved hairs (Fig. 5G). Telson elongate, triangular, with rounded posterior apex in both sexes, bearing abundant setae and slender, curved hairs as on subanal plate (Fig. 6D, E).

**Male**: typical scutigerid gonopod styles on first and second genital segment (Figs 5H, 6A). Length of first gonopod 1.5× second gonopod. Both pairs of gonopods covered with setae and dense hairs. Subanal plate with parabolic outline, relatively shorter than in female, non-setose band along middle of subanal plate (Fig. 5F).

##### Etymology.

The specific epithet *kaijiangensis* is derived from the locality Kaijiang, Sichuan Province, China, referring to the type locality of the species.

#### ﻿ Thereuopoda edgecombei

Taxon classificationAnimalia

Ji, Liu & Zhang sp. nov.

BDA85577-21D1-56A3-BBE4-685D3181A8E5

https://zoobank.org/6B1AD2C2-F8E3-461C-B8DB-1AF7AB36002A

**Figure 8.**
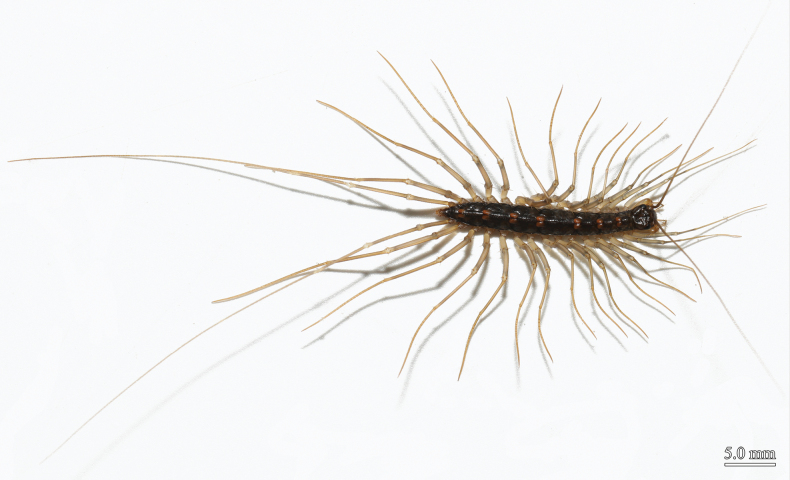
*Thereuopoda edgecombei* sp. nov., paratype female. Scale bars: 5.0 mm.

**Figure 9.**
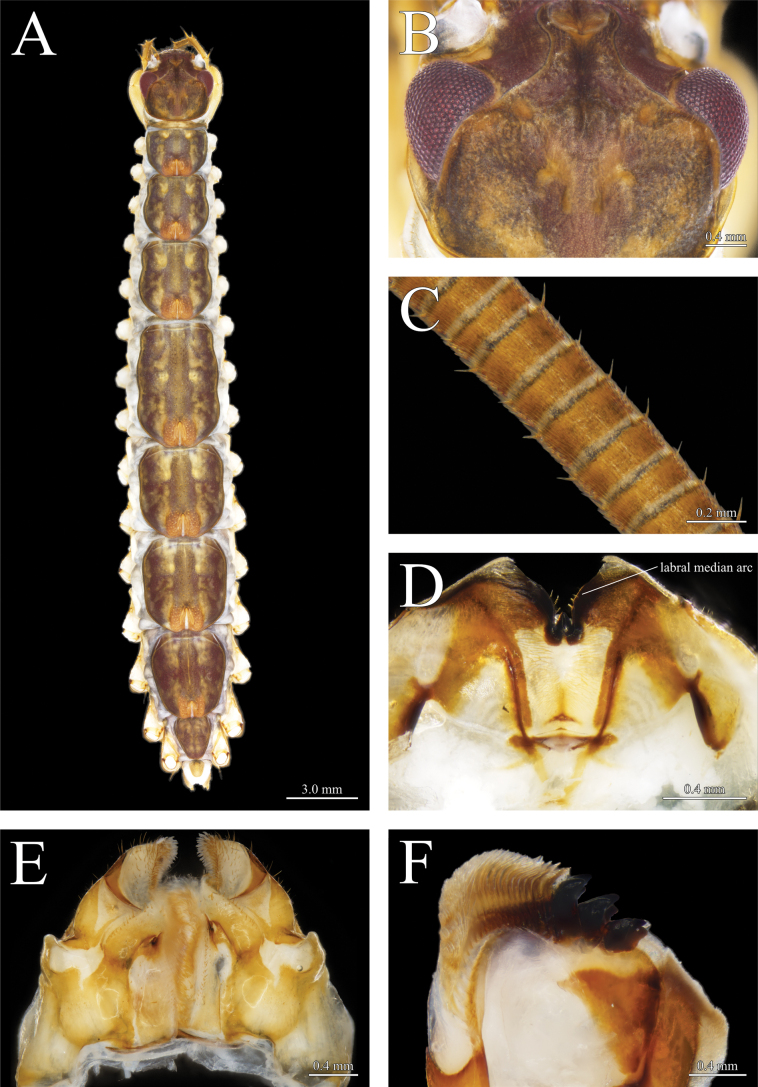
*Thereuopoda edgecombei* sp. nov., holotype male. **A.** Habitus, dorsal view; **B.** Head capsule; **C.** First flagellum of antenna; **D.** Epipharynx; **E.** First maxillae; **F.** Mandible. Scale bars: 3.0 mm (**A**); 0.4 mm (**B, D–F**); 0.2 mm (**C**).

**Figure 10.**
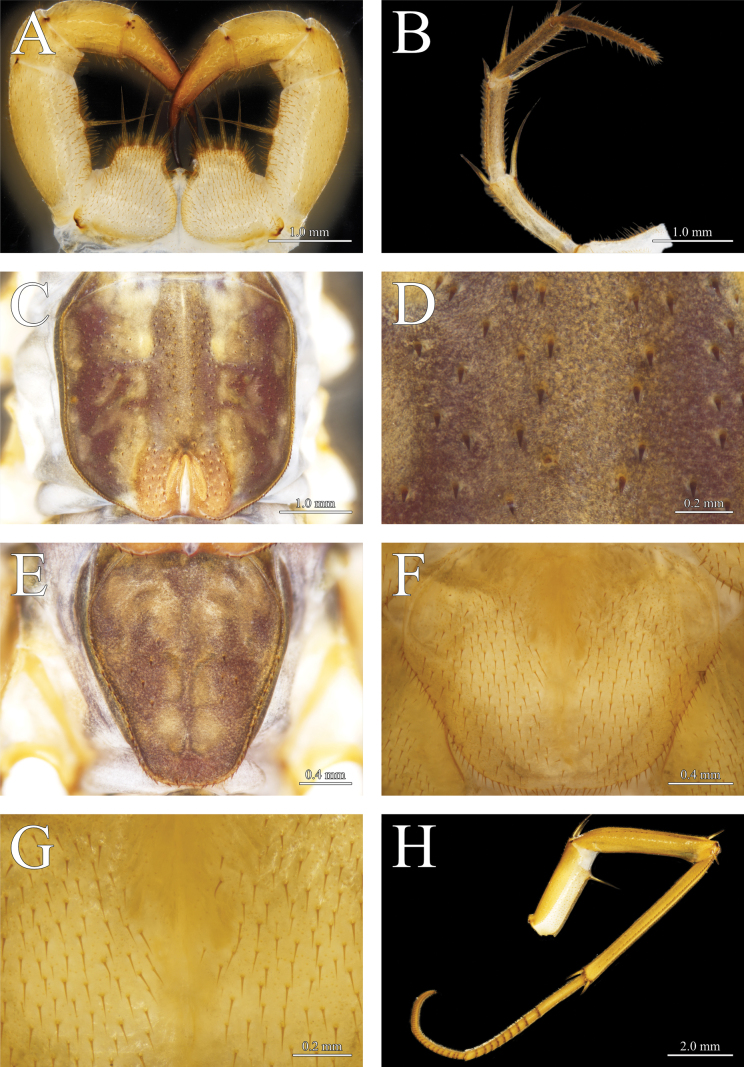
*Thereuopoda edgecombei* sp. nov., holotype male. **A.** Forcipular segment; **B.** Second maxilla; **C.** Tergite 6; **D.** Details of tergite 6; **E.** Tergite 8; **F.** Sternite 15; **G.** Details of sternite 15; **H.** Leg 10. Scale bars: 1.0 mm (**A–C**); 0.2 mm (**D, G**); 0.4 mm (**E, F**); 2.0 mm (**H**).

**Figure 11.**
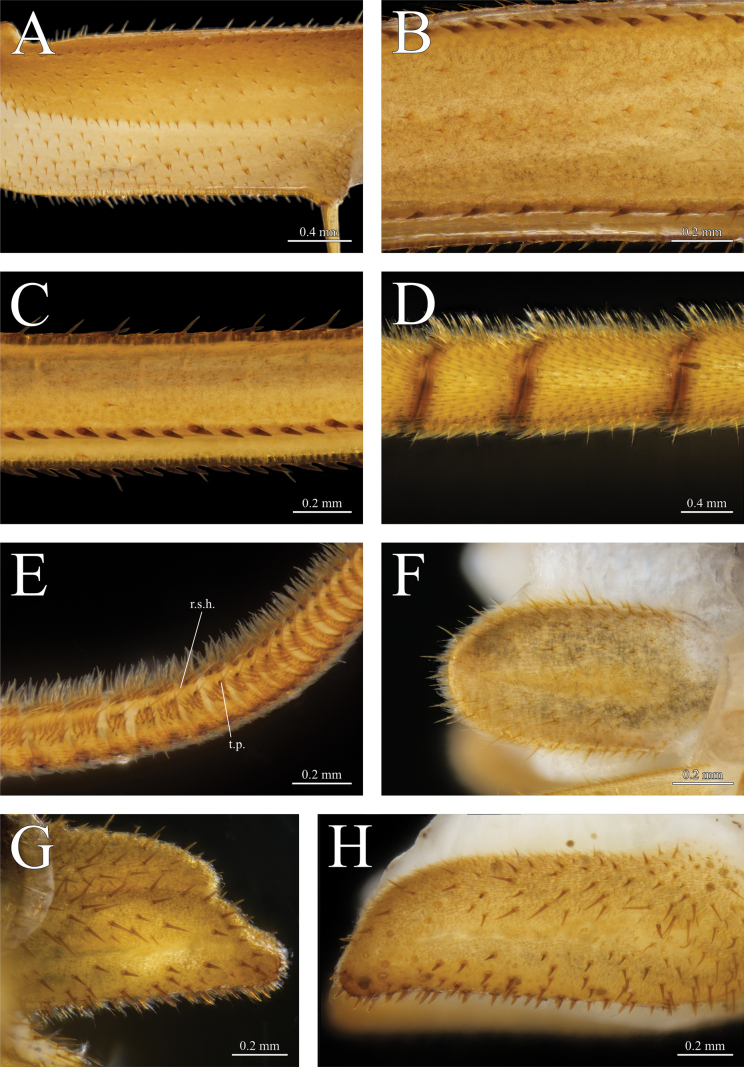
*Thereuopoda edgecombei* sp. nov., holotype male. **A.** Leg 10 prefemur; **B.** Leg 10 femur; **C.** Leg 10 tibia; **D.** Leg 10 tarsus I; **E.** Leg 10 tarsus II, ventral view, showing resilient sole hairs (r.s.h.) and tarsal papillae (t.p.); **F.** Male subanal plate. Paratype female. **G.** Female subanal plate; **H.** Female subanal plate. Scale bars: 0.4 mm (**A, D**); 0.2 mm (**B, C, E–H**).

**Figure 12.**
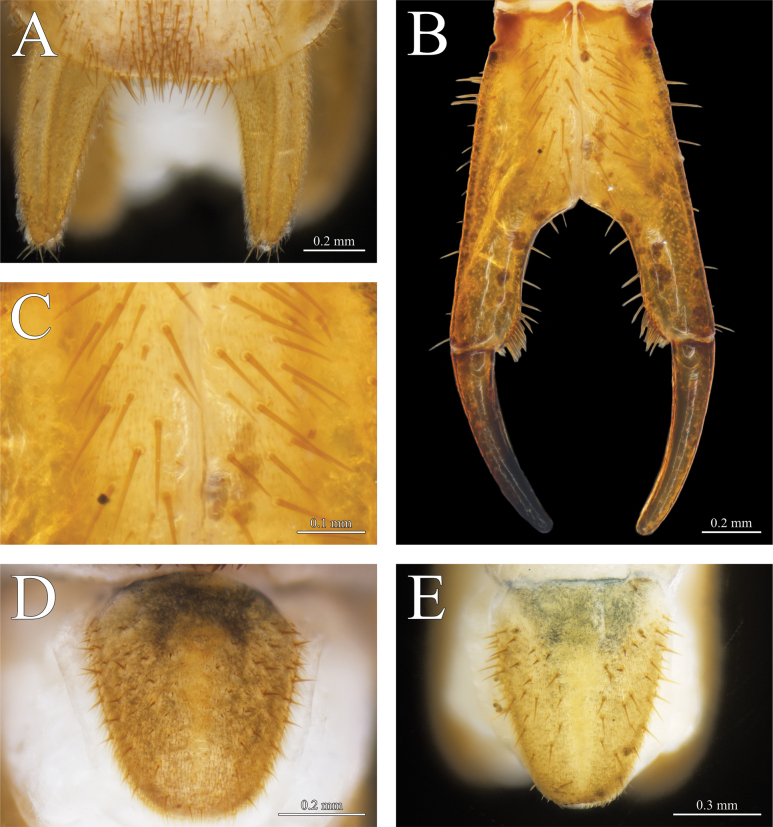
*Thereuopoda edgecombei* sp. nov., holotype male. **A.** Male gonopod; **B.** Female gonopod; **C.** Proarthron of female gonopod; **D.** Male telson. Paratype male. **E.** Female telson. Scale bars: 0.2 mm (**A, B, D**); 0.1 mm (**C**); 0.3 mm (**E**).

**Figure 13.**
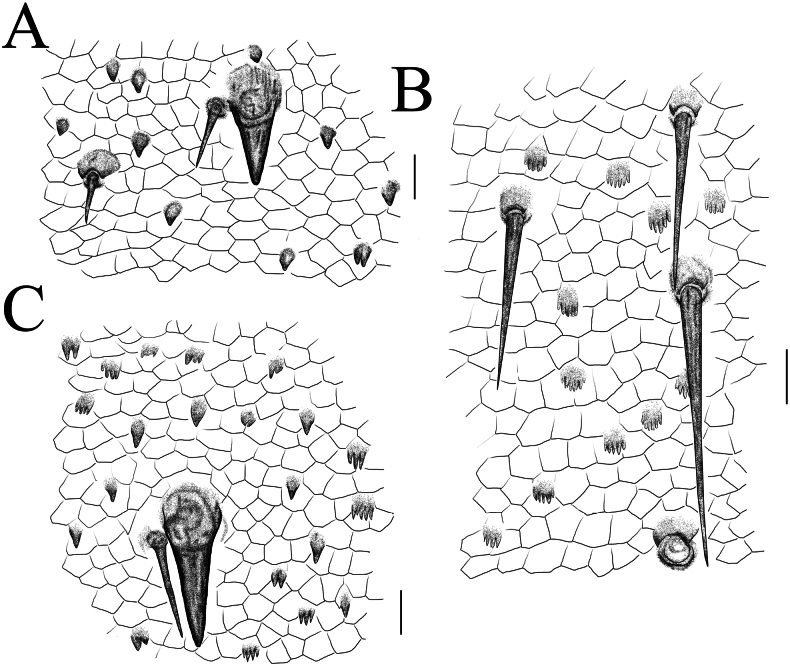
*Thereuopoda edgecombei* sp. nov., paratype female. **A.** Tergite 6. **B.** Tergite 8. **C.** Sternite 15. Scale bars: 20 μm.

Figs 8, 9, 10, 11, 12, 13; Tables 3, 4

##### Type material.

***Holotype*.** China • Male; Hainan Province, Chengmai County, Dafeng Town; 19°51'N, 110°2'E; 19 Aug. 2024; Jiehong Ji leg.; ZJNU HNCM33. ***Paratype*.** China • 2 females, 1 male; same data as for holotype; ZJNU HNCM32, HNCM34, HNCM117.

##### Diagnosis.

*Thereuopoda* with body length up to 31 mm (Fig. 8). Head capsule yellow with dark-brown medial patch; radiating network of dark-brown pigment in the posterolateral part of head capsule (Fig. 9A, B). Tergites pale yellow with three longitudinal chestnut-brown stripes; transverse projections between central and lateral stripes; a narrow yellow stripe in the middle of central stripe (Figs 9A, 10C). Stoma saddles orange. Posterior margin of tergites unevenly rounded. Spiracle on T6 about 60–70% length of the stoma saddle. Posterior margin of T8 without median embayment (Fig. 10E). Leg 15 1.79–1.94× body length. Tibial spine-bristles 0/1 on leg 1. Maximum length of female gonopod 2.03–2.37× maximum width (Fig. 12B). Hairs on middle area of proarthron (Fig. 12C); angle at median distal end of proarthron about 100°. Sinus between mesarthron parabolic, broad with apex distinctly or weakly pointed (Fig. 12B). Female subanal plate posterior termination pointed or with process (Fig. 11G, H). Length of first male gonopod and male second gonopod subequal (Fig. 12A).

##### Description.

Length up to 29 mm in largest female, 31 mm in largest male.

**Colour**: head capsule yellow with dark-brown medial patch; radiating network of dark-brown pigment on posterolateral part of head capsule (Fig. 9A, B). Compound eye dark purple. Tergites pale yellow, with three longitudinal chestnut-brown stripes; transverse projections between central and lateral stripes; a narrow yellow stripe in middle of central stripe (Figs 9A, 10C). Stoma saddles orange. Leg yellow-orange with grey or blue-green pigment at the end of femur (Fig. 10H). Sternites and female gonopod yellow (Figs 10F, 12B).

**Head capsule**: anterior projection of sutures with divergent posterior part; lateral edge blunt and rounded (Fig. 9B). Posteromedian impression moderately deep. Antenna 1.14–1.64× body length. First flagellum with 49–61 articles; ring-like articles with dense hairs and a few setae forming a single whorl surrounding distal end of article (Fig. 9C).

**Epipharynx**: labral median arc 1.82–2.17× depth of labral mid-piece tooth (Fig. 9D).

**Mandible**: three teeth, all smooth-surfaced and tricuspid (Fig. 9F). Nearly 21 pectinate lamellae on gnathal edge.

**First maxilla**: inner margin of distal article of telopodite with dense, brush-like setae (Fig. 9E). Short, simple setae on ventral surface of coxal process.

**Second maxilla**: prefemur with dorsal and ventral spine-bristles; femur with four spine-bristles; tibia with two dorsal spine-bristles (Fig. 10B).

**Forcipular segment**: coxa nearly as long as wide, with four subequal, long, spine-bristles on anterior margin (Fig. 10A).

**Tergites**: posterior margin of tergites unevenly rounded (Fig. 10C). Borders of TT6–8 with heavy spines in saw-like fringe. Length: median-wide ratio in T4 1.38–1.57. Stoma saddles strongly vaulted. Spiracles very long, spiracle on T6 about 60–70% length of stoma saddle. Posterior margin of T8 without median embayment (Fig. 10E). Number of spines on stoma saddles and tergites as shown in Table 3. Unpaired large spines on TT2–8, each paired with *Tastborsten* (Figs 10D, 13A, B). Isolated *Tastborsten* with short, paired spines at bases on all tergites; these spines joined at a common base. Basal spines 10–20% as long as associated bristle. All tergites with spiculae and spinulae. Short, triangular spiculae separated by several polygonal scales that lack spiculae.

**Table 3.** Number of spines on stoma saddle and whole tergites of *Thereuopoda edgecombei* sp. nov.

Tergites	Stoma saddles	Whole tergites
1	0	0
2	1–9	9–14
3	25–39	71–132
4	43–66	260–401
5	48–72	185–303
6	46–54	160–282
7	26–31	89–184
8	–	2–9

**Legs**: tarsus I and II segmentation as shown in Table 4. Leg 15 length 1.79–1.94× that of body. Prefemoral spine-bristles in a 2/1 pattern on legs 1–15; femoral spine-bristles 1/2 on all legs; tibial spine-bristles 0/1 on leg 1, 0/1 or 0/2 on leg 2, 1/2 on leg 3–15. Spine rows on prefemur, femur and tibia with spine–setae pairing (Fig. 11A–C). Tarsus I of leg 6–14 with spines (Fig. 11D). Tarsus II of legs 1–14 with paired and equally sized tarsal papillae on successive segments except for first and last few segments on each leg (Fig. 11E). Resilient sole hairs originating near posteromedial edge of tarsal papilla, extending to the succeeding segment. Dense tuft of setae on ventrolateral side of tarsus II.

**Table 4.** Number of segments in Tarsus I and Tarsus II of *Thereuopoda edgecombei* sp. nov.

Leg	Tarsus I	Tarsus II
1	14–20	42–47
2	13–16	41–44
3	12–14	40–45
4	11–14	35–41
5	12–14	32–37
6	10–18	28–37
7	9–11	32–36
8	10–13	31–39
9	10–12	34–37
10	11–12	37–39
11	10–14	34–44
12	12–18	30–44
13	11–13	36–47
14	13–15	40–51

**Sternites**: median embayment in posterior margin of sternites lacking or shallow (Fig. 10F). Longitudinal median furrow observable passing through entire length of the sternites. Minute, multifurcating spinulae and scattered setae with paired short spines in all sternites (Figs 10G, 13C).

**Female**: gonopod with maximum length 2.03–2.37× maximum width. Longitudinal median suture in proarthron complete (Fig. 12B). Lateral margins of proarthron and mesarthron divergent posteriorly. Subtriangular depression on proarthron without setae. Hairs and setae on middle area of proarthron (Fig. 12C). Angle at median distal end of proarthron about 100°. Proarthron 1.18–1.42× length of mesarthron. Distomedial corner of mesarthron with a cluster of 12–15 setae. Sinus between mesarthron parabolic, broad with apex distinctly or weakly pointed. Width of mesarthron 0.54–0.66× maximum width of sinus. Proarthron + mesarthron 1.77–1.87× length of metarthron. Outer margin of metarthron uniformly curved. Subanal plate with ventral margin straighter than curved dorsal margin, posterior termination pointed or with process; maximum length 2.69–3.31× maximum height; smooth, non-setose band along middle of subanal plate; setae of varied size on outer surface of subanal plate between which are slender, curved hairs (Fig. 11G, H). Telson elongate, triangular, with rounded posterior apex in both sexes, bearing abundant setae and slender, curved hairs as on subanal plate (Fig. 12D, E).

**Male**: typical scutigerid gonopod styles on first and second genital segment (Fig. 12A). Length of first gonopod and male second gonopods subequal. Both pairs of gonopods covered with setae and dense hairs. Subanal plate with parabolic outline, relatively shorter than in female, non-setose band along middle of subanal plate (Fig. 11F).

##### Etymology.

The species is dedicated to Dr Gregory D. Edgecombe, in recognition of his pioneering contributions to the taxonomy and phylogeny of Scutigeromorpha and for his generous provision of critical literature on this group.

### ﻿Genetic distance and phylogenetic relationships of two new species

**Figure 14.**
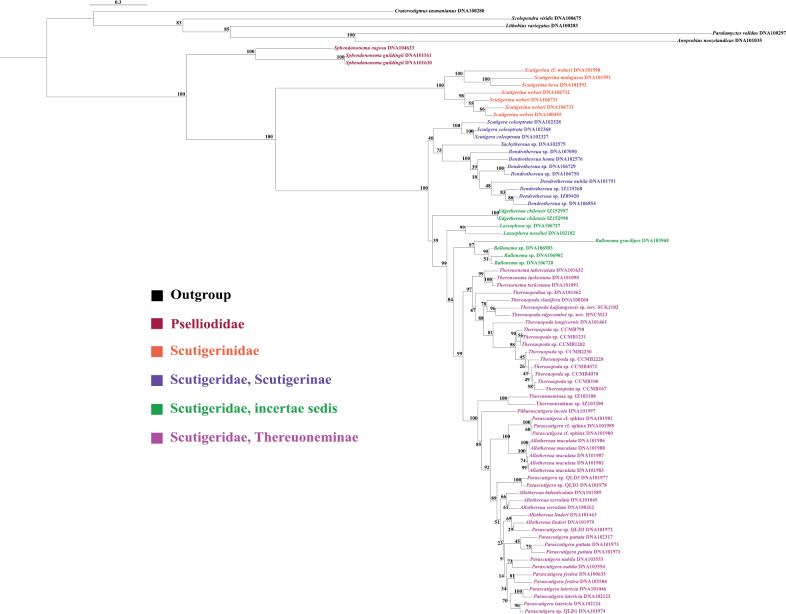
Phylogenetic relationships of Scutigeromorpha were inferred from ML analyses based on six genes (18S rRNA, 28S rRNA, H3, 16S rRNA, 12S rRNA, and COX1).

The genetic distances among the five *Thereuopoda* species based on COX1 ranged from 13% to 18% (Suppl. material 3). The lowest interspecific distance was observed between *T. edgecombei* sp. nov. and *T. kaijiangensis* sp. nov., amounting to 13%. The phylogenetic analyses revealed an initial divergence between Pselliodidae and the remaining taxa, followed by a major split separating Scutigerinidae from Scutigeridae. Within Scutigeridae, further resolution indicated a primary division between Scutigerinae and Thereuoneminae, although monopoly of these two subfamilies was not supported. Within *Thereuopoda*, the ML and BI trees showed identical topologies (Fig. 14, Suppl. material 4): (((*T. edgecombei* sp. nov. + *T. kaijiangensis* sp. nov.) + *T. clunifera*) + (*T. longicornis* + *T.* sp.)).

## ﻿Discussion

Two new species, *Thereuopoda kaijiangensis* sp. nov. and *T. edgecombei* sp. nov., can be distinguished from each other based on diagnostic characters listed in Table 5. Both of these new species can be easily distinguished from the morphologically similar *T. longicornis* by the relatively greater length of the female gonopod (2.25–2.6× in *T. kaijiangensis*, 2.03–2.37× in *T. edgecombei* versus 1.3× in *T. longicornis*) (Krishnan and Prasad 2022). Additionally, *T. kaijiangensis* and *T. edgecombei* exhibit a radiating network of dark-brown pigment on the posterolateral part of the head capsule, as opposed to dark greenish-brown patches in *T. longicornis*. There are three longitudinal stripes on central and lateral tergites in *T. kaijiangensis* and *T. edgecombei*, which are in contrast to a single brownish median longitudinal stripe in *T. longicornis*. Both ML and BI analyses recovered the monophyly of *Thereuopoda*, and the congruent topology, branch lengths, and the genetic distance provide robust evidence for the establishment of the two new species.

**Table 5.** Comparison of *Thereuopoda* species from China.

Characters	*T. kaijiangensis* sp. nov.	*T. edgecombei* sp. nov.	*T. longicornis*	*T. clunifera*
Pigment in the posterolateral part of head capsule	Radiating network	Radiating network	Patch	–
Stripes on tergites	Three longitudinal stripes	Three longitudinal stripes	One median longitudinal stripe	Cross–shaped stripe
Posterior margin of tergites	Unevenly rounded	Unevenly rounded	Unevenly rounded	Evenly rounded
Median embayment in posterior margin of T8	Present	Absent	–	–
Tibial spine-bristles on leg 1	0/2	0/1	–	–
Length of Leg 15 / body	2.85–3.14	1.79–1.94	–	–
A/B of female gonopod	2.25–2.6	2.03–2.37	1.3	–
Hairs on proarthron of female gonopod	Absent	Present	–	–
Shape of posterior termination of female subanal plate	Pointed or with process	Pointed or with process	Pointed or with process	Blunt and rounded
Length of first male gonopod / second male gonopod	1.5	Subequal	–	–

Three poorly known taxa—T. (P.) chinensis, *Scutigera complanata* Haase, 1887 and *Scutigera sinuata* Haase, 1887—require comparison with the two new species. Thereuopoda (P.) chinensis was originally described based on a single female specimen collected from Macao, and its taxonomic validity remains uncertain (Verhoeff 1905; Würmli 1979). According to Verhoeff’s original description, T. (P.) chinensis exhibits a greyish-yellow median longitudinal stripe (“Rücken nur mäßig gewölbt, grün, mit graugelblicher, auch über die Sättel ausgedehnter Mittelbinde”) and lacks spines on femora of legs 1–4 (“Femur des 1.–4. B. unbedornt”). The generic placement of *S. complanata* and *S. sinuata* remains uncertain, and both species lack longitudinal stripes on the tergites (“Ohne deutliche Längsstreifen auf den Schilden”) (Haase 1887). In contrast, the two new species possess three median longitudinal stripes and have spines on femora of all legs. They are clearly distinct from these three previously described taxa and are not considered conspecific with any of them. A major challenge in advancing the taxonomy and phylogeny of scutigeromorph centipedes is that numerous species were originally described based on limited material, with no additional data available since their initial descriptions (Butler et al. 2010). It is anticipated that this group will receive increased taxonomic and systematic attention in the future.

### ﻿Key to known *Thereuopoda* species in China

**Table:** 

1	Posterior border of tergites evenly rounded	***T. clunifera***
–	Posterior border of tergites unevenly rounded	**2**
2	One median longitudinal stripe on tergites	***T. longicornis***
–	Three longitudinal stripes on tergites	**3**
3	Hairs absent on proarthron of female gonopod	***T. kaijiangensis* sp. nov.**
–	Hairs present on proarthron of female gonopod	***T. edgecombei* sp. nov.**

## Supplementary Material

XML Treatment for Thereuopoda kaijiangensis

XML Treatment for Thereuopoda edgecombei

## ﻿Additional information

### Use of AI

No use of AI was reported.

### Author ORCIDs

Jie-Hong Ji https://orcid.org/0009-0001-9381-0306

Hui-Yuan Wu https://orcid.org/0009-0007-6064-4819

Dan-Na Yu https://orcid.org/0000-0002-9870-1926

Jia-Yong Zhang https://orcid.org/0000-0002-7679-2548

### Data availability

All of the data that support the findings of this study are available in the main text or Supplementary Information.

## Supplementary materials

Supplementary material 1 List for each sequenced locus Data type pdf Explanation note List of taxa, numbers, localities, and GenBank accession numbers for each sequenced locus. https://binary.pensoft.net/file/1496810This dataset is made available under the Open Database License (http://opendatacommons.org/licenses/odbl/1.0/). The Open Database License (ODbL) is a license agreement intended to allow users to freely share, modify, and use this Dataset while maintaining this same freedom for others, provided that the original source and author(s) are credited.Jie-Hong Ji, Chen-Yang Shen, Yi-Xin Gao, Hui-Yuan Wu, Dan-Na Yu, Sheng-Long Liu, Jia-Yong Zhang

Supplementary material 2 Scheme and best-fitting models Data type pdf Explanation note Best partitioning scheme and best-fitting models selected by PartitionFinder v. 2.2.1. https://binary.pensoft.net/file/1496811This dataset is made available under the Open Database License (http://opendatacommons.org/licenses/odbl/1.0/). The Open Database License (ODbL) is a license agreement intended to allow users to freely share, modify, and use this Dataset while maintaining this same freedom for others, provided that the original source and author(s) are credited.Jie-Hong Ji, Chen-Yang Shen, Yi-Xin Gao, Hui-Yuan Wu, Dan-Na Yu, Sheng-Long Liu, Jia-Yong Zhang

Supplementary material 3 Genetic distances Data type pdf Explanation note Genetic distances of *Thereuopoda* species based on COX1. https://binary.pensoft.net/file/1496812This dataset is made available under the Open Database License (http://opendatacommons.org/licenses/odbl/1.0/). The Open Database License (ODbL) is a license agreement intended to allow users to freely share, modify, and use this Dataset while maintaining this same freedom for others, provided that the original source and author(s) are credited.Jie-Hong Ji, Chen-Yang Shen, Yi-Xin Gao, Hui-Yuan Wu, Dan-Na Yu, Sheng-Long Liu, Jia-Yong Zhang

Supplementary material 4 Phylogenetic trees Data type tiff Explanation note Phylogenetic relationships of Scutigeromorpha were inferred from Bayesian-inference analyses based on six genes (18S rRNA, 28S rRNA, H3, 16S rRNA, 12S rRNA, and COX1) https://binary.pensoft.net/file/1496813This dataset is made available under the Open Database License (http://opendatacommons.org/licenses/odbl/1.0/). The Open Database License (ODbL) is a license agreement intended to allow users to freely share, modify, and use this Dataset while maintaining this same freedom for others, provided that the original source and author(s) are credited.Jie-Hong Ji, Chen-Yang Shen, Yi-Xin Gao, Hui-Yuan Wu, Dan-Na Yu, Sheng-Long Liu, Jia-Yong Zhang
