# Supplementary material for: ﻿Two new species of the genus Thereuopoda Verhoeff, 1904 (Scutigeromorpha, Scutigeridae) from Sichuan and Hainan Provinces, China
Source: Zookeys. 2025 Dec 19;1264:351–76. doi: 10.3897/zookeys.1264.165241 (PMC12743254; doi:10.3897/zookeys.1264.165241)
Supplement: Supplementary material 1 — List for each sequenced locus [file zookeys-1264-351_article-165241__-s001.pdf]

Table S1. List of taxa, numbers, localities, and GenBank accession numbers for each sequenced locus.

Taxon	Number	Location	18S rRNA	28S rRNA	16S rRNA	COI	H3	12S rRNA
Outgroups								
Lithobius variegatus rubriceps	DNA100283		AF000773	AF000780	AY084071	AF334311	–	–
Anopsobius neozelanicus	DNA101035		AF173248	DQ222132	AF334337	DQ222165	DQ222179	–
Paralamyctes validus	DNA100297		AF334289	AF334310	AF334357	AF334329	DQ222180	–
Craterostigmus tasmanianus	DNA100280		AF000774	DQ222133	AF370860	AF370835	AF110850	–
Scolopendra viridis	DNA100675		DQ201419	DQ222134	DQ201425	DQ201431	DQ222181	–
Pselliodidae								
Sphendononema guildingii	DNA101161	Brazil	DQ222121	DQ222138	–	–	DQ222185	–
Sphendononema guildingii	DNA101630	Brazil	DQ222122	DQ222139	DQ222154	DQ222168	DQ222186	FJ660871
Sphendononema rugosa	DNA104623	Cameroon	HQ591445	HQ591446	HQ591448	HQ591450	HQ591451	HQ591453
Scutigerinidae								
Scutigerina cf. *weberi*	DNA101590	Madagascar	DQ222118	–	DQ222151	DQ222166	DQ222182	FJ660870
Scutigerina hova	DNA101592	Madagascar	DQ222120	DQ222137	DQ222153	–	DQ222184	–
Scutigerina malagassa	DNA101591	Madagascar	DQ222119	KF218758	DQ222152	DQ222167	DQ222183	FJ660869
Scutigerina weberi	DNA100455	Swaziland	AY288689	AY288705/ DQ222135	AY288717	AY288741	AY428835	–
Scutigerina weberi	DNA106731	South Africa	KF218741	KF218759	KF218776	KF218788	KF218801	KF218811
Scutigerina weberi	DNA106732	South Africa	KF218742	KF218760	KF218777	–	KF218802	KF218813
Scutigerina weberi	DNA106733	South Africa	KF218743	KF218761	–	KF218789	–	KF218812
Scutigeridae, Scutigerinae								
Scutigera coleoptrata	DNA102327	Turkey	FJ660707	FJ660748	FJ660788	–	FJ660844	FJ660876
Scutigera coleoptrata	DNA102328	Georgia	FJ660708	FJ660749	–	FJ660819	FJ660845	FJ660877
Scutigera coleoptrata	DNA102368	Bulgaria	FJ660711	FJ660752	FJ660791	FJ660820	FJ660848	FJ660880
Tachythereua sp.	DNA102575	Senegal	FJ660716	FJ660756	FJ660795	–	FJ660852	FJ660884

Dendrothereua homa	DNA102576	Arizona, USA	FJ660705	FJ660746	FJ660786	FJ660818	FJ660842	FJ660873
Dendrothereua nubila	DNA101791	Costa Rica	FJ660704	FJ660744	FJ660785	FJ660817	FJ660841	FJ660872
Dendrothereua sp.	DNA106729	Mexico	KF218744	KF218765/KF218766	KF218778	KF218790	KF218803	–
Dendrothereua sp.	DNA106730	Mexico	KF218745	KF218767	KF218779	KF218791	–	–
Dendrothereua sp.	DNA106854	Mexico	KF218746	KF218768	–	KF218792	–	–
Dendrothereua sp.	DNA107050	Dominican Republic	KF218747	KF218769	KF218780	–	KF218804	–
Dendrothereua sp.	IZ-125268	Nicaragua	KF218749	KF218771/KF218772	–	KF218793	–	–
Dendrothereua sp.	IZ-89420	Guatemala	KF218748	KF218770	KF218781	KF218794	–	–
Scutigeridae, incertae sedis								
Ballonema gracilipes	DNA103968	Papua New Guinea	KF218750	–	HQ591449	–	HQ591452	HQ591454
Ballonema sp.	DNA106728	Papua New Guinea	KF218751	KF218762	KF218782	KF218796	KF218805	–
Ballonema sp.	DNA106902	Papua New Guinea	KF218752	KF218763	KF218783	KF218795	KF218806	KF218814
Ballonema sp.	DNA106903	Papua New Guinea	KF218753	KF218764	KF218784	KF218797	KF218807	KF218815
Lassophora nossibei	DNA102102	Madagascar	FJ660714	FJ660755	FJ660794	FJ660821	FJ660851	FJ660883
Lassophora sp.	DNA106727	Mozambique	KF218754	KF218773	KF218785	KF218798	KF218808	KF218816
Edgethereua chilensis	IZ-152997	Chile	PP086649	PP086651	PP086653	PP086508	–	PP086655
Edgethereua chilensis	IZ-152998	Chile	PP086650	PP086652	PP086654	–	–	–
Scutigeridae, Thereuoneminae								
Allothereua bidenticulata	DNA101589	NSW, Australia	FJ660717	FJ660757	FJ660796	FJ660822	–	FJ660885
Allothereua linderi	DNA101463	NSW, Australia	DQ222128	DQ222147	DQ222160	DQ222174	DQ222195	FJ660886
Allothereua linderi	DNA101979	NSW, Australia	FJ660718	FJ660758	FJ660797	–	FJ660853	FJ660887
Allothereua maculata	DNA101982	WA, Australia	FJ660720	FJ660759	FJ660798	FJ660823	FJ660854	FJ660888
Allothereua maculata	DNA101983	WA, Australia	FJ660721	FJ660760	FJ660799	FJ660824	FJ660855	FJ660889
Allothereua maculata	DNA101986	WA, Australia	FJ660722	FJ660761	FJ660800	FJ660825	FJ660856	FJ660890

Allothereua maculata	DNA101987	WA, Australia	FJ660723	FJ660762	FJ660801	FJ660826	–	FJ660891
Allothereua maculata	DNA101988	WA, Australia	FJ660724	FJ660763	FJ660802	FJ660827	FJ660857	FJ660892
Allothereua serrulata	DNA100262	NSW, Australia	DQ222129	DQ222148	DQ222161	DQ222175	DQ222197	FJ660893
Allothereua serrulata	DNA101045	QLD, Australia	DQ222130	FJ660764	DQ222162	DQ222176	DQ222198	–
Parascutigera cf. *sphinx*	DNA101980	WA, Australia	FJ660740	FJ660780	FJ660813	FJ660838	FJ660866	FJ660905
Parascutigera cf. *sphinx*	DNA101981	WA, Australia	FJ660741	FJ660781	FJ660814	FJ660839	FJ660867	FJ660906
Parascutigera cf. *sphinx*	DNA101985	WA, Australia	FJ660739	FJ660779	–	FJ660837	FJ660865	FJ660904
Parascutigera festiva	DNA100635	New Caledonia	AY288688	DQ222149	DQ222163	DQ222177	DQ222199	FJ660894
Parascutigera festiva	DNA102584	New Caledonia	FJ660725	FJ660766	FJ660803	FJ660828	FJ660858	FJ660895
Parascutigera guttata	DNA101971	QLD, Australia	FJ660727	FJ660768	FJ660805	–	–	–
Parascutigera guttata	DNA101973	QLD, Australia	FJ660728	FJ660769	FJ660806	–	–	–
Parascutigera guttata	DNA102317	QLD, Australia	FJ660726	FJ660767	FJ660804	FJ660829	FJ660859	FJ660896
Parascutigera latericia	DNA101046	New Caledonia	DQ222131	DQ222150	DQ222164	DQ222178	DQ222200	–
Parascutigera latericia	DNA102123	New Caledonia	FJ660730	FJ660770	FJ660807	FJ660830	FJ660860	–
Parascutigera latericia	DNA102124	New Caledonia	FJ660731	FJ660771	–	FJ660831	–	FJ660897
Parascutigera nubila	DNA103553	New Caledonia	KF218755	FJ660772	FJ660808	FJ660832	–	FJ660898
Parascutigera nubila	DNA103554	New Caledonia	FJ660733	FJ660773	–	FJ660833	–	FJ660899
Parascutigera sp. QLD1	DNA101974	QLD, Australia	FJ660735	FJ660775	FJ660809	FJ660834	FJ660861	FJ660900
Parascutigera sp. QLD2	DNA101972	QLD, Australia	FJ660736	FJ660776	FJ660810	–	FJ660862	FJ660901
Parascutigera sp. QLD3	DNA101977	QLD, Australia	FJ660737	FJ660777	FJ660811	FJ660835	FJ660863	FJ660902
Parascutigera sp. QLD3	DNA101978	QLD, Australia	FJ660738	FJ660778	FJ660812	FJ660836	FJ660864	FJ660903
Pilbarascutigera incola	DNA101997	WA, Australia	FJ660742	FJ660782	FJ660815	–	FJ660868	FJ660907
Thereuoneminae sp.	IZ-103188	Guam	KF218756	KF218774	KF218786	KF218799	KF218809	–
Thereuoneminae sp.	IZ-103200	Micronesia	KF218757	KF218775	KF218787	KF218800	KF218810	–
Thereuonema tuberculata	DNA101632	Japan	DQ222126	DQ222145	DQ222158	DQ222173	DQ222193	FJ660908
Thereuonema turkestana	DNA101090	Uzbekistan	FJ660743	FJ660784	FJ660816	FJ660840	–	FJ660909

Thereuonema turkestana	DNA101091	Uzbekistan	DQ201417	DQ222144	DQ201423	DQ201427	DQ222192	–
Thereuopoda clunifera	DNA100260	Japan	AF173239	DQ222142	AY288716	DQ222171	DQ222190	FJ660910
Thereuopoda longicornis	DNA101461	Thailand	DQ222125	DQ222143	DQ222157	DQ222172	DQ222191	–
Thereuopoda sp.	CCMB100	Lakhari Valley WLS	PP874140	PP874071	PP874012	–	PP868432	–
Thereuopoda sp.	CCMB167	Karlapat WLS	PP874141	PP874072	PP874013	–	PP868433	–
Thereuopoda sp.	CCMB798	Ranebennur WLS	PP874165	PP874095	PP874032	–	PP868456	–
Thereuopoda sp.	CCMB1202	Gundlabramhmeswaram WLS	PP874178	PP874108	PP874044	–	PP868468	–
Thereuopoda sp.	CCMB1231	Gundlabramhmeswaram WLS	PP874179	PP874109	PP874045	–	PP868469	–
Thereuopoda sp.	CCMB2228	Radhanagari WLS	PP874199	PP874128	PP874061	–	PP868486	–
Thereuopoda sp.	CCMB2230	Radhanagari WLS	PP874201	PP874130	PP874063	–	PP868488	–
Thereuopoda sp.	CCMB4070	Odisha Eastern Ghats	PP874137	PP874068	PP874009	–	PP868429	–
Thereuopoda sp.	CCMB4072	NISER Campus, Jatni	PP874207	PP874136	PP874067	PP882778	PP868490	–
Thereuopoda edgecombei sp. nov.	HNCM23	Hainan, China	PV816238	PV816250	PQ595912	PQ595912	PV817827	PQ595912
Thereuopoda kaijiangensis sp. nov.	SCKJ102	Sichuan, China	PV816239	PV816241	PV822024	PV815842	PV817828	PV822025
Thereuopodina sp.	DNA101462	QLD, Australia	DQ222127	DQ222146	DQ222159	–	DQ222194	FJ660911
