# Supplementary material for: ﻿Two new species of the genus Thereuopoda Verhoeff, 1904 (Scutigeromorpha, Scutigeridae) from Sichuan and Hainan Provinces, China
Source: Zookeys. 2025 Dec 19;1264:351–76. doi: 10.3897/zookeys.1264.165241 (PMC12743254; doi:10.3897/zookeys.1264.165241)
Supplement: Supplementary material 2 — Scheme and best-fitting models [file zookeys-1264-351_article-165241__-s002.pdf]

Table S2. Best partitioning scheme and best-fitting models selected by PartitionFinder v.2.2.1.

**Subset**	**Subset partitions**	**Best model**
1	COX1, 12S rRNA, 16S rRNA, 28S rRNA	GTR+I+G
2	18S rRNA	TRNEF+I+G
3	H3	HKY+I+G
