# Supplementary material for: ﻿Two new species of the genus Thereuopoda Verhoeff, 1904 (Scutigeromorpha, Scutigeridae) from Sichuan and Hainan Provinces, China
Source: Zookeys. 2025 Dec 19;1264:351–76. doi: 10.3897/zookeys.1264.165241 (PMC12743254; doi:10.3897/zookeys.1264.165241)
Supplement: Supplementary material 3 — Genetic distances [file zookeys-1264-351_article-165241__-s003.pdf]

Table S3. Genetic distances of *Thereuopoda* species based on COX1.

	T. clunifera DNA100260	*T. longicornis* DNA101461	*T. edgecombei* sp. nov.	*T. kaijiangensis* sp. nov.	*T.* sp. CCMB4072
T. clunifera DNA100260					
T. longicornis DNA101461	0.17				
T. edgecombei sp. nov.	0.17	0.17			
T. kaijiangensis sp. nov.	0.15	0.15	0.13		
T. sp. CCMB4072	0.18	0.17	0.18	0.16	
