# Supplementary figures and images for: ﻿Two new species of the genus Thereuopoda Verhoeff, 1904 (Scutigeromorpha, Scutigeridae) from Sichuan and Hainan Provinces, China
Source: Zookeys. 2025 Dec 19;1264:351–76. doi: 10.3897/zookeys.1264.165241 (PMC12743254; doi:10.3897/zookeys.1264.165241)

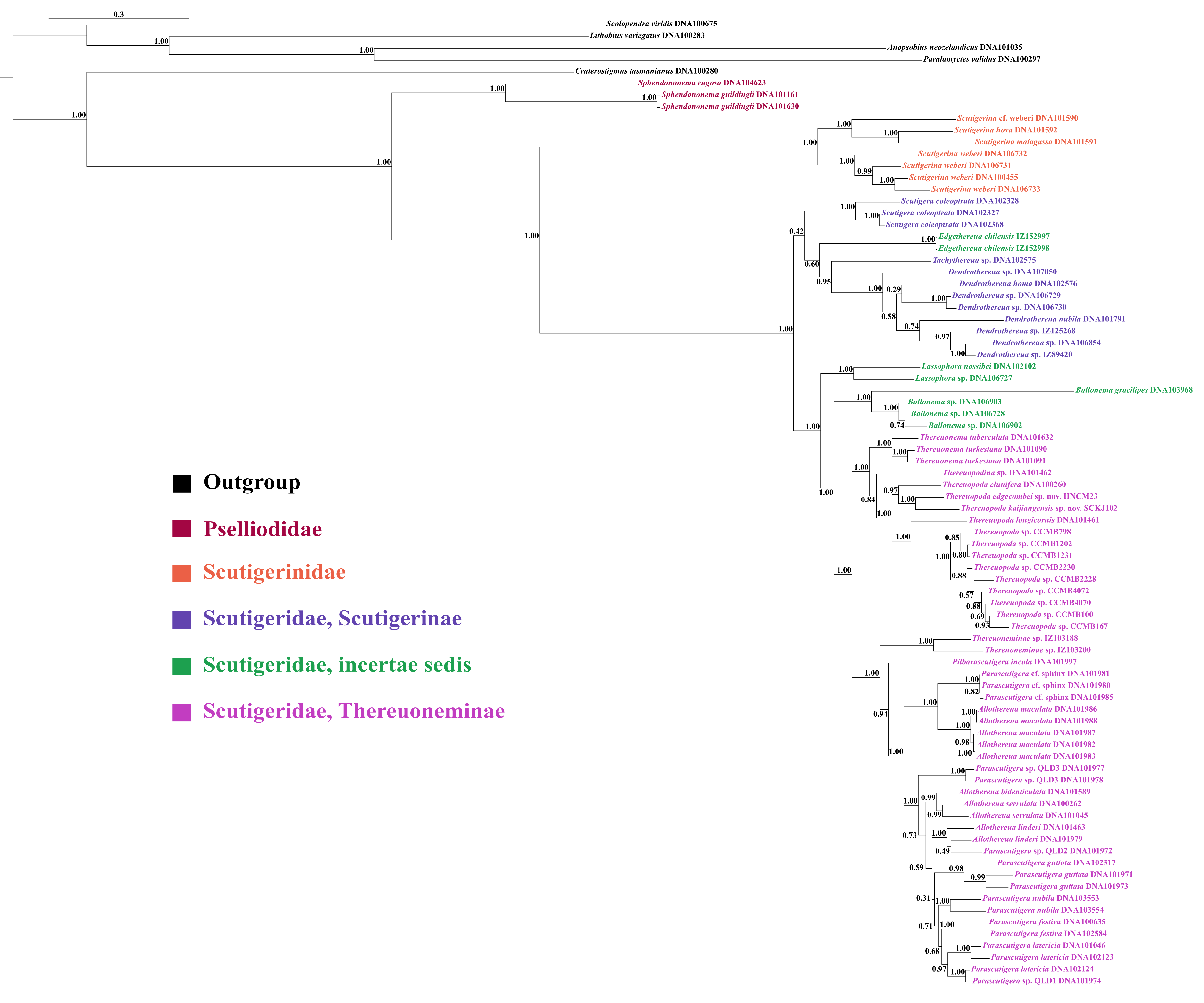

Supplement: Supplementary material 4 — Phylogenetic trees [file zookeys-1264-351_article-165241__-s004.tiff]
